# The moderating role of SES on genetic differences in educational achievement in the Netherlands

**DOI:** 10.1038/s41539-019-0052-2

**Published:** 2019-09-03

**Authors:** Eveline L. de Zeeuw, Kees-Jan Kan, Catharina E. M. van Beijsterveldt, Hamdi Mbarek, Jouke-Jan Hottenga, Gareth E. Davies, Michael C. Neale, Conor V. Dolan, Dorret I. Boomsma

**Affiliations:** 1 Department of Biological Psychology, Vrije Universiteit, Amsterdam, the Netherlands; 2 Amsterdam Public Health Research Institute, VUmc, Amsterdam, the Netherlands; 3 College of Child Development and Education, University of Amsterdam, Amsterdam, the Netherlands; 4 Qatar Genome Programme, Qatar Foundation, Doha, Qatar; 5 Avera Institute for Human Genetics, Avera McKennan Hospital & University Health Center, Sioux Falls, SD USA; 6 Department of Psychiatry, Virginia Commonwealth University, Richmond, VA USA; 7 Department of Human and Molecular Genetics, Virginia Commonwealth University, Richmond, VA USA

**Keywords:** Molecular neuroscience, Human behaviour

## Abstract

Parental socioeconomic status (SES) is a strong predictor of children’s educational achievement (EA), with an increasing effect throughout development. Inequality in educational outcomes between children from different SES backgrounds exists in all Western countries. It has been proposed that a cause of this inequality lies in the interplay between genetic effects and SES on EA, which might depend on society and the equality of the education system. This study adopted two approaches, a classical twin design and polygenic score (PGS) approach, to address the effect of parental SES on EA in a large sample of 12-year-old Dutch twin pairs (2479 MZ and 4450 DZ twin pairs with PGSs for educational attainment available in 2335 children) from the Netherlands Twin Register (NTR). The findings of this study indicated that average EA increased with increasing parental SES. The difference in EA between boys and girls became smaller in the higher SES groups. The classical twin design analyses based on genetic covariance structure modeling pointed to lower genetic, environmental, and thus phenotypic variation in EA at higher SES. Independent from a child’s PGS, parental SES predicted EA. However, the strength of the association between PGS and EA did not depend on parental SES. In a within-family design, the twin with a higher PGS scored higher on EA than the co-twin, demonstrating that the effect of the PGS on EA was at least partly independent from parental SES. To conclude, EA depended on SES both directly and indirectly, and SES moderated the additive genetic and environmental components of EA. Adding information from PGS, in addition to parental SES, improved the prediction of children’s EA.

## Introduction

Parental socioeconomic status (SES), based on education level, occupation and income, is one of the strongest predictors of children’s educational achievement (EA).^1^ Children in high SES families score higher on EA, and this SES-related gap increases throughout development, from a difference of ~0.5 SD at age 5 (parents without high school diploma versus parents with a college degree) increased to ~1 SD at age 17.^2^ These findings apply to all key school subjects, including reading, mathematics,^3^ language^4^, and spelling.^5^ In the Netherlands, low EA is related to short-term negative outcomes such as delinquency^6^ and school drop-out^7^ in adolescents. Long-term outcomes are a lower income, higher crime rate in adulthood^8^ and a markedly lower life expectancy.^9^ Multiple causes of such inequality in educational outcomes between children in low and high SES families have been proposed. There may be direct causal effects of SES on EA, because high SES parents generally have more resources to invest in, and so to further, the EA of their offspring. Another cause of this inequality might be that high SES parents may both create a positive learning environment for their children, and transmit to their biological offspring genes that directly or indirectly facilitate EA.^10^

There is a positive interrelationship of general cognitive ability, SES, and educational attainment and a well-established heritability of general cognitive ability^11^ and educational achievement.^12^ Intelligence is an important determinant of EA, which predicts level of occupation, and consequently income. The three main indicators of socioeconomic status, educational attainment, occupation and income, also show influences of genetic differences between people.^13–15^ and correlate at the genetic level^15^ due to their dependence on both general intelligence, and on other relevant heritable traits (e.g., personality, neurodevelopmental traits, such as hyperactivity and inattention). Consequently, individual differences in parental SES, which is defined primarily in terms of educational attainment, occupation and income, are partly genetic. Given these findings there is likely to be some degree of genotype-SES correlation, i.e., a child’s genotype is associated with parental SES. We do not suggest that this association is purely genetic as environmental influences are likely to contribute too.

Previous research in the United Kingdom has shown that the higher General Certificate of Secondary Education (GCSE) scores of children attending private versus public schools may be partly due to differences in genetic influences between the children from different SES groups.^16^ In some countries (US), but not others (Western European countries and Australia), genetic variance in EA, as measured with cognitive test scores standardized within each dataset, was higher in high SES children (0.61 versus 0.24), while common and unique environmental variance was higher in low SES children, but this difference was not significant.^17^ It has been suggested that this is due to the fact that children from high SES families have more opportunities to develop their genetic potential while genetic differences in children from low SES are suppressed because of their environmental circumstances. However, a recent study based on birth and school records from all children in Florida found no evidence for SES-related differences in genetic variance,^18^ indicating that even within countries differential effects are observed. The difference in the size of genetic and environmental variance components of EA across SES, suggests the presence of genotype-by-SES interaction, i.e., SES moderates the effect of genetic and environmental influences.

Studies investigating the role of SES on the effects of genes relevant to EA have traditionally focused on the moderation by SES of genetic and environmental variance components.^18,19^ Developments in molecular genetic studies now provide the opportunity to also investigate this at the level of measured individual genetic variants (genetic loci that display individual differences). A genome-wide association study (GWAS) tests the association between a variant and a phenotype in a population, at millions of genetic loci, which thus requires a stringent significance level. Usually a series of GWAS studies is combined in a meta-analysis. An effect size is estimated for each genetic loci and these effect sizes can be employed to construct polygenic scores (PGS) for individual subjects. The score reflects a combined effect for a number of selected loci with a trait. This set can include all, also non-significant, loci or selected loci based on a *p*-value threshold. For each individual in a target sample, which is independent from the GWAS sample, a PGS is calculated by multiplying the number of effect alleles per loci (0, 1, or 2) with the effect size, summed over all loci in the considered set of loci.^20,21^ A recent GWAS (EA3) in over 1 million individuals identified 1271 genome-wide significant loci on autosomal chromosomes associated with the number of years of schooling completed.^22^ Nearly all genetic variants involved in educational attainment have small effects, but their collective effects (PGS) explains a substantial proportion of the genetic variation (11–13%).^22^ PGSs based on genetic variants from a GWA study for educational attainment in ~300,000 individuals (EA2),^23^ provided a measure of children’s genetic potential with respect to EA in a recent study from the United Kingdom.^24^ The PGS for educational attainment was significantly related to SES in a sample of 16-year-olds. Children with higher PGS came from higher SES families on average, which supports the presence of genotype-by-SES correlation. On the other hand, variance in EA due to the genetic effect of the PGS on EA did not vary over SES levels, i.e., there was no evidence for genotype-by-SES interaction.

Almost all previous studies of the interaction between SES and genetic effects on educational achievement have been conducted in samples from the United States and United Kingdom. However, in these countries educational systems are decentralized and are characterized by large differences in educational opportunities.^25^ Private schools, unlike state schools, require potentially prohibitive tuition fee and select students based on entrance exam results. Therefore, the present study investigates the interplay between genes and SES in relation to EA in the Netherlands, a more egalitarian country, where nearly all schools are state-supported and adhere to the same governmentally imposed standards and curriculum, regardless of whether they are private or state schools.

The aim of the current study was to explore the effects of SES on EA, in a large sample of 12-year-old twins from the Netherlands Twin Register (NTR). We adopted two approaches, genetic covariance structure modeling in a classical twin design, and polygenic score analyses, to quantify the interplay between parental SES and children’s EA. First, we assessed the moderating effect of SES on the mean of EA and on the genetic and environmental variance components underlying differences in EA (within-family design). Second, we examined if (i) the magnitude of the genetic variance, as captured by a PGS, depended on SES, (ii) the association between PGS and EA differed across SES groups (between-family design) and (iii) the DZ twin with the higher PGS scored higher on EA than their co-twin with the lower PGS, to determine the effect of a child’s PGS on EA while accounting for the effect of parental SES on EA (within-family design).

## Results

### Descriptives

**Fig. 1:**
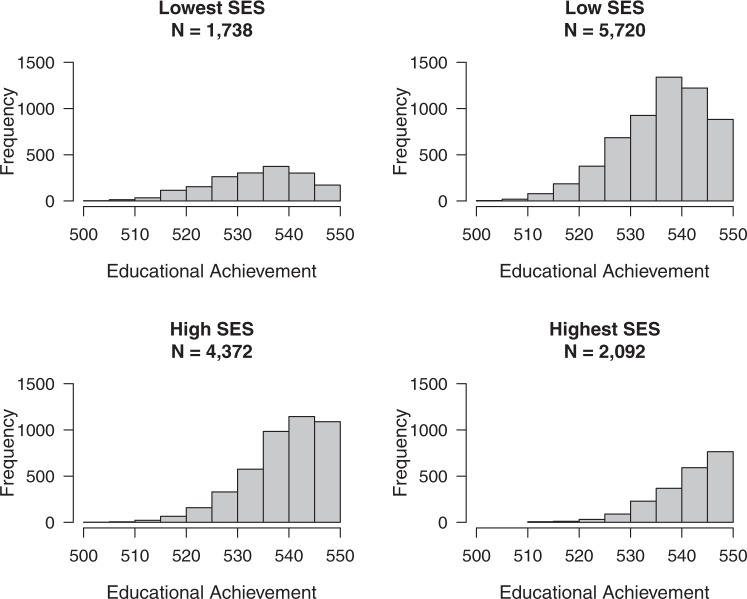
Frequency of the educational achievement (EA) scores in 12-year-olds for each socioeconomic status (SES) group

**Fig. 2:**
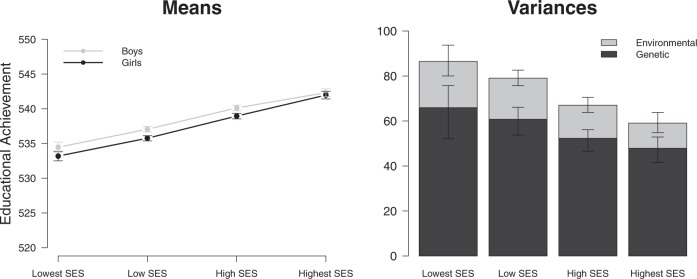
The means and (unstandardized) genetic and environmental (common and unique) variances (and their 95% confidence intervals) of the educational achievement (EA) scores in 12-year-olds for each socioeconomic status (SES) group

**Fig. 3:**
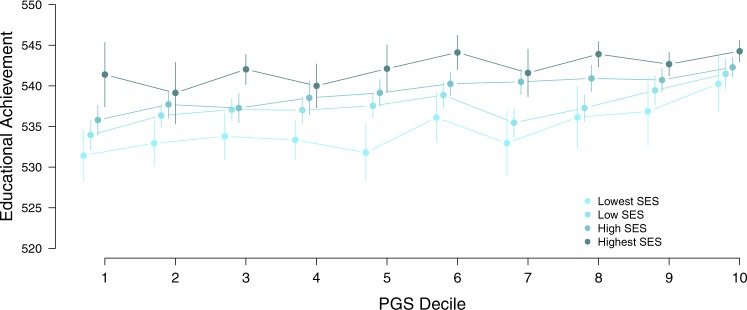
The means (and their 95% confidence intervals) of the educational achievement (EA) scores in 12-year-olds (*N* = 2335) for each PGS decile and separately for each socioeconomic status (SES) group

**Fig. 4:**
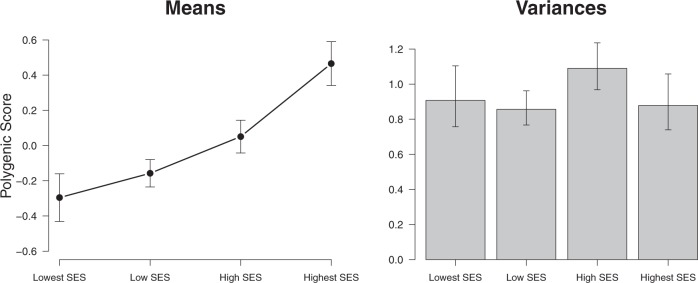
The means and variances (and their 95% confidence intervals) of the polygenic score (PGS) for educational attainment in 12-year-olds for each socioeconomic status (SES) group

There were 320 MZ and 571 DZ twin pairs in the lowest SES group, 1030 MZ and 1859 DZ twin pairs in the low SES group, 766 MZ and 1378 DZ twin pairs in the high SES group, and 363 MZ and 642 DZ twin pairs in the highest SES group. Figure 1 gives the distribution of the EA scores in each of these SES groups. The subsequent analyses were corrected for censoring as there was more restriction of range in the higher SES groups, in which more children obtain the maximum score of 550. Figure 2 displays the means and (unstandardized) genetic and environmental (common and unique) variances of EA in each SES group. EA depended on SES in multiple ways. As expected, the higher the SES, the higher, on average, the mean EA. Boys had a significantly higher EA than girls in the lowest (*Δ*_*−2LL*_ = 7.0, *Δ*_*df*_ *=* 1, *p* = 0.008), low (*Δ*_*−2LL*_ = 26.7, *Δ*_*df*_ *=* 1, *p* < 0.001) and high (*Δ*_*−2LL*_ = 19.5, *Δ*_*df*_ *=* 1, *p* *<* 0.001) SES group, but not in the highest (*Δ*_*−2LL*_ = 0.9, *Δ*_*df*_ *=* 1, *p* = 0.346) SES group. Figure 3 displays the means and standard deviations of the EA test score for each of the PGS deciles and separately for SES group. Figure 4 depicts the mean and variances of the PGS score across the SES groups. In higher SES groups, children had, on average, a higher PGS, meaning that they inherited more alleles with a positive effect on educational attainment (*Δ*_*−2LL*_ = 81.3, *Δ*_*df*_ *=* 3, *p* < 0.001). The variance in PGS was not consistently moderated by SES as it could be equated across the SES groups (*Δ*_*−2LL*_ = 9.2, *Δ*_*df*_ *=* 3, *p* = 0.026).

### Genetic covariance structure modeling

In the total sample twin correlations were 0.79 (MZ) and 0.40 (DZ) and individual differences in EA were highly heritable (72%). The common environmental variance (8%) was attributable to SES effects as shown by the non-significant contribution of the C variance component after controlling for SES (*Δ*_*−2LL*_ = 0.0, *Δ*_*df*_ *=* 1, *p* = 0.963). The twin correlations, corrected for censoring, were very similar across the different SES groups (lowest SES: *r*_MZ_ = 0.79, *r*_DZ_ = 0.40; low SES: *r*_MZ_ = 0.79, *r*_DZ_ = 0.40; high SES: *r*_MZ_ = 0.78, *r*_DZ_ = 0.39; highest SES: *r*_MZ_ = 0.81, *r*_DZ_ = 0.41). Table 1 shows the estimated means and variances of each SES group. Noticeably, genetic and environmental variance differed across the groups and depended on SES (*Δ*_*−2LL*_ = 89.7, *Δ*_*df*_ *=* 9, *p* < 0.001). Specifically, the genetic and environmental variance components decreased with increasing SES.

**Table 1:** Means and variance components (and their 95% confidence intervals) of the educational achievement (EA) scores in 12-year-olds for each socioeconomic (SES) group as estimated in the genetic covariance structure models

	Lowest SES *N* = 891 twin pairs	Low SES *N* = 2889 twin pairs	High SES *N* = 2144 twin pairs	Highest SES *N* = 1005 twin pairs
Means				
Girls	533.2 (532.5–533.9)	535.8 (535.4–536.1)	538.9 (538.6–539.3)	542.0 (541.4–542.5)
Boys	534.5 (533.7–535.2)	537.0 (536.7–537.4)	540.1 (539.7–540.5)	542.3 (541.8–542.9)
Unstandardized variances				
Phenotypic	86.5 (80.0–93.7)	79.1 (75.7–82.6)	67.0 (63.8–70.5)	59.0 (54.7–63.7)
Genetic	65.9 (52.1–75.8)	60.8 (53.7.–66.1)	52.3 (46.6–56.1)	47.8 (41.5–52.8)
Common environmental	2.7 (0.0–15.8)	1.8 (0.0–8.3)	0.0 (0.0–4.9)	0.0 (0.0–5.4)
Unique environmental	17.9 (15.2–21.2)	16.5 (15.0–18.1)	14.7 (13.2–16.4)	11.2 (9.6–13.2)
Proportion of phenotypic variance				
Genetic	0.76 (0.60–0.83)	0.77 (0.68–0.81)	0.78 (0.70–0.80)	0.81 (0.77–0.84)
Common environmental	0.03 (0.00–0.18)	0.02 (0.00–0.10)	0.00 (0.00–0.07)	0.00 (0.00–0.09)
Unique environmental	0.21 (0.17–0.25)	0.21 (0.19–0.23)	0.22 (0.20–0.25)	0.19 (0.16–0.23)

### Polygenic score analyses

In the between-family analysis, the regression coefficient in the association of educational achievement on PGS did not differ between the SES groups (*Δ*_*−2LL*_ = 2.3, *Δ*_*df*_ = 3, *p* = 0.517), which means that the strength of the effect of genetic variants related to educational attainment in predicting EA in children did not differ between SES groups. The intercepts differed between SES groups (*Δ*_*−2LL*_ = 61.8, *Δ*_*df*_ = 3, *p* < 0.001), showing that there were mean differences in EA across SES groups independent from differences in PGS between children. In the DZ twin pairs the correlation for the PGSs (*r* = 0.55 (95% CI 0.49–0.61)) was slightly higher than its expected value of 0.5, possible reflecting assortative mating. The DZ twins with the higher PGS than their co-twin (mean difference of ~0.75 SD) scored significantly higher (~1.5 points) on the EA test (*Δ*_*−2LL*_ = 11.4, *Δ*_*df*_ = 1, *p* < 0.001), indicating that PGS was associated with EA independently of SES. The within-family analysis showed that there was an association between PGS and EA that is not confounded by possible stratification across SES.

## Discussion

As expected, parental SES was found to be strongly associated with EA on a standardized test taken at the end of primary school. Boys scored higher than girls in all SES groups, except in the highest SES group where there was no sex difference in EA. It has been suggested that sex differences in test scores are due to voluntary outside-of-class experiences, which possibly are more similar in boys and girls from high SES families.^26^ To a large extent, differences between children in EA were attributable to genetic differences. SES accounted for a small, but significant, portion of the common environmental variance in EA. In the higher SES groups, the phenotypic variance in EA was smaller and this could be attributed to a decrease in both the genetic and the environmental variance component with increasing SES. An interaction between a child’s genotype and the environment in which the child is raised may contribute to the difference in genetic and environmental variance between SES groups. In this case, alleles associated with lower educational achievement seem to have larger detrimental effects in children that grow up under less advantageous social and economic circumstances. This is in line with the previous finding that results in the Netherlands are absent or even reversed compared with those found in the United States,^17^ specifically, the lower genetic variance in high SES families.^27^

DNA methylation has been suggested as one mechanism through which the environment causes variation in the genetic effects on educational attainment and as such could be a source of phenotypic variance.^28^ Epigenome-wide association (EWAS) studies looking at differential methylation identified several cytosine guanine dinucleotides (CpG; a CG base pair at a single DNA strand of that is linked by a phosphate site, thereby influencing gene expression), in genes involved in neuronal, immune and developmental processes. Methylation levels were associated with educational attainment after adjusting for lifestyle factors, including smoking status. This points to the possibility that there are (causal) epigenetic consequences due to differential environmental exposures across SES, for example maternal smoking during pregnancy, air pollution, and maternal folate levels.^29,30^

The PGS, based on genetic variants association with educational attainment in adults, was associated with parental SES with a higher mean PGS in the children from high SES families compared with children from low SES families. The variance of this PGS did not differ between SES groups. When predicting EA in the children from their PGS, the PGS for educational attainment was positively associated with EA in all SES groups, but the strength of this relationship was not moderated by SES. We note that the percentage of variance explained by the PGS is limited by the GWAS on which they are based and as such only reflects part of the genetic variance involved in EA. In addition, the PGS is more likely to capture genetic variants that have a homogenous effect across multiple populations and environments, compared with effects of genetic variants that are sensitive to moderation by SES, resulting in a limited power to detect an interaction between genotype and SES.

The association between PGS and EA might be confounded by a genotype-SES correlation, i.e., children who grow up in higher SES families have, on average, a higher PGS. In the current study, we addressed this potential confounding by employing a within-family design. In families with DZ twin offspring, the twin with the higher PGS scored higher on the EA compared with the co-twin with the lower PGS. This demonstrates that a child’s PGS was related to EA even when taking into account the possible genotype-SES correlation confounding effect of parental SES as SES is shared by siblings growing up in the same household. Sibling differences in PGS have also been found to predict differences in EA in cohorts from the United States, United Kingdom, and New Zealand.^31^ The significant effect of the PGS in a within-family design tells us that there also is a direct effect of genetic differences in educational attainment on children’s EA.

Although parental SES is a strong predictor of children’s EA, there are large differences between children within each SES group, especially in the lower SES groups. The EA scores in the lowest SES group displayed the full range from the lowest to the highest possible score. This was not the case in the highest SES group, where the lowest score was absent, while many children achieve the highest score. More importantly, even in relatively egalitarian societies SES still has an effect when taking into account the effect of genetic differences between people, as also observed in other societies than the Netherlands. Two recent studies from Australia and Iceland employed a virtual parent design to partly disentangle the causal effects of the home environment and a child’s genotype. A non-transmitted PGS reflects the part of parental genotypes that is not inherited by a child, as parents both transmit 50% of their genome to their offspring. The non-transmitted PGS had a significant effect on a child’s EA above the effect of a child’s own PGS and this effect disappeared when controlling for parental SES.^32,33^

A limitation of this study is the higher percentage of children from a high SES background in our sample. In the Netherlands ~30% of the adults have a college degree which suggests that the parental SES in our sample is not completely representative of all Dutch primary school children.^34^ The percentage of children who obtained the highest educational achievement score was larger in the higher SES groups. The lower total variance in the higher SES group was partly due to ceiling effects.^35^ Nevertheless, correcting for the effect of censoring at the high end of the distribution by using a procedure that was verified in simulations yielded the same results.

Differences between children from the same SES group were partly due to differences in genetic predisposition. As a child’s PGS is measured at the level of the individual child instead of the family it has added value to SES in predicting which children are at risk for lower EA. Identification of these children is key as they are at double risk because of their family background and their disadvantageous genetic predisposition. Policies to enhance EA could in the future focus on increasing the mean level of EA in these children through, for example, extra educational programs, to reduce inequality in educational outcomes and to ensure that all children are able to develop the academic skills that are needed to succeed in society.

## Methods

### Participants

The NTR, which was established in 1987, is a population-based register that recruits multiples and their family members for longitudinal research.^36^ Parents of twins receive a survey about the development of their children every 2 to 3 years until the twins are 12 years old. From age 7 onward, parents are asked for consent to approach the teachers who rate the development of the twins and their siblings. Results on the standardized EA test were collected from parents, teachers, and from the children themselves. Written informed consent was obtained from parents and the data collection was approved by the medical ethical review committee of the VU Medical Center Amsterdam (NTR25052007). More details concerning the NTR’s data collection, the methods of recruitment, participants’ background and response rates are described elsewhere.^36^

Data on EA at age 12 and data on parental SES were available in 12,889 twins with known zygosity from 6929 families (6123 boys and 6766 girls; birth cohorts 1979–2002). The sample included data from 5959 complete twin pairs with known zygosity and 970 (14.0%) incomplete twin pairs (2479 MZ and 4450 DZ twin pairs). Data on EA may be missing because the decision to administer the test lies with each school, and because children who had to repeat a grade may have yet to take the test at the time of data collection. PGSs for educational attainment were available for 2335 children with genome-wide single-nucleotide polymorphism (SNP) data. Among the children with PGS data there were 496 complete DZ twin pairs.

### Measures

Educational achievement (EA) was based on a national EA test, which consists of multiple-choice items. The test measures scholastic knowledge, including language and mathematical skills.^37,38^ Around 75% of Dutch children take this test in their final year of primary school (around age 12) on 3 consecutive days in February (before 2015) or April (from 2015 onward). The total scores were converted into standardized scores by comparing a child with the total group of children who take the test in that school year (e.g., in 2015 ~165,000 children). A linear transformation based on item-response-theory (IRT) analyses was implemented to equate the test to previous versions. The total scores were not normalized before standardization and, as a result, the standardized scores show a negatively skewed distribution with a minimum of 501, a maximum of 550, a mean of 535 and a standard deviation of 9. The internal reliability (*α* *=* 0.95) and the test-retest reliability of the test are good (*r* *=* 0.96).^39^ Non-response analysis revealed that parents from a lower SES were less likely to return the information on EA. However, differences, while statistically significant, were small.^36^

SES was based on a combination of information on parental current job status, occupational level and education level. Current job status reflected if the parent was currently employed or not due to either being a student, being incapacitated or being unemployed. Occupational level was either derived from a detailed description of the parental occupation, which was classified according to the Standard Classification of Occupations,^40^ or obtained using the Erikson-Goldthorpe (EGP)-classification scheme.^41^ Coding of the occupations was based on the mental complexity of the work and ranged from low skilled to scientific work. Education level was based on the highest level of education followed/attained. If a person’s current job status was unemployed he/she was categorized into SES level 1, irrespective of occupational and educational level. Allocation of the people with a current job to SES level 1–5 was based on a combination of occupational and educational level. For example, someone who works in middle management with an educational level of higher vocational education completed or higher was classified as SES level 4, but if he/she had an educational level lower than higher vocational educational completed he/she was classified as SES level 3. In light of the relatively small number of families falling in the first parental SES level, the two lowest SES levels were collapsed, resulting in four SES groups (*N*_Lowest_: 12.9%, *N*_Low_: 41.7%, *N*_High_: 30.9%, *N*_Highest_: 14.5%). Parental SES was assessed when the twins were aged 3, 7, and 9/10 years old and based on the highest parental score within a family.

Genotype data were assessed on Illumina and Affymetrix platforms and cross-platform imputed against the Genome of the Netherlands (GoNL) reference set.^42^ For the quality control (QC) of the samples, criteria were: genotype call rate > 0.90, heterozygosity −0.10 < *F* < 0.10, no mismatch for gender between known status and genotypic assessment, and not an ethnic outlier.^43^ QC criteria of the single-nucleotide polymorphisms (SNP) were minor allele frequency (MAF) > 0.01, Hardy-Weinberg equilibrium (HWE) *p* > 10^−5^, call rate > 0.95, Mendelian error rate < 20 and, for palindromic SNPs, MAF < 0.40 and > 0.50. After cross-platform imputation, SNPs were included when HWE *p* > 0.00001, imputation quality *R*^2^ > 0.90, and Mendelian error rate < mean + 3 SD. Phasing and imputation was performed using MaCH-Admix software.^44^

### Statistical analyses

Model fitting was conducted in the statistical program R, version 3.4.4^45^ using package OpenMx version 2.9.9.^46^ Parameters were estimated by raw data full information maximum likelihood. A censoring correction was implemented to account for differences in ceiling effects between the SES groups. To accommodate the censoring the likelihood function was based explicitly on the censored bivariate normal distribution with fixed censoring threshold at the maximum value of 550.^35,47^ In all models, sex was included as a covariate, to allow for sex differences in mean EA.

### Genetic covariance structure modeling

The first step involved fitting a classical univariate twin model to decompose phenotypic differences in EA into variance due to (additive) genetic variance (G), shared or common environmental variance (C), and unique or non-shared environmental variance (E).^48^ The classical twin design relies on the difference in genetic resemblance between monozygotic (MZ) and dizygotic (DZ) twin pairs. MZ twin pairs share all of their alleles identically by descent from their parents (barring the negligible effects of postzygotic mutations).^49^ DZ twin pairs share, on average, 50% of their alleles identically by descent. We tested whether the common environmental variance component explained a significant proportion of the variance in EA by comparing a model with and without, i.e., variance fixed to zero, this variance component. In a next step, a model in which mean EA values were stratified by SES was fitted to the data. This model was compared with the previous model to determine whether the common environmental variance was due to differences in SES between children. In this model the presence of sex differences in the mean of EA for each SES group was also tested. Subsequently, both means and variance components were estimated stratified by SES. This model was compared with the model in which only means were estimated separately across SES groups to assess whether the A, C, and E variance components were moderated by SES.

### Polygenic score analyses

PGSs were calculated using best guess genotype data by multiplying the number of effect alleles with the corresponding regression weight of each measured genetic variant (i.e., single-nucleotide polymorphism (SNP)), from the most recent GWA meta-analysis of educational attainment.^22^ They were based on results excluding NTR, to avoid an overestimation of associations,^50^ and excluded 23andMe data, as we did not have permission to use these data. SNPs that passed quality control were used to construct PGSs in LDpred.^51^ The PGSs were corrected for population stratification by regressing out principal components (10 Dutch PCs and 10 global PCs) reflecting ancestry differences and corrected for the type of array used for genotyping.^43^

**Fig. 5:**
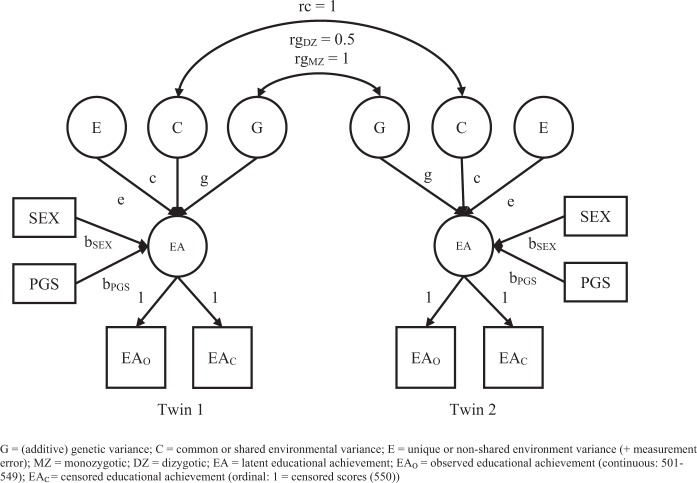
Graphical representation of the classical twin model, as it was estimated in each socioeconomic status (SES) group, with the educational achievement (EA) scores in 12-year-olds regressed on sex (and polygenic score (PGS) for educational attainment)

In a first model, in a between-family design, the means and variances of the PGS were estimated stratified by SES and compared with models in which either the means or the variances were equated across SES groups to test mean differences in PGS across SES and to test whether the magnitude of the genetic variance was moderated by SES. Subsequently, within each SES group, EA was regressed on a child’s PGS and sex, to accommodate sex differences in mean EA, in the twin design (with the correction for censoring (see Fig. 5)). In this model we estimated the effect of the educational attainment PGS on a child’s EA within each SES group. The residual variance was decomposed into genetic and environmental variance components. To explore whether the strength of the relationship between EA and the PGS was moderated by SES, we tested whether the regression coefficient for PGS was equal across SES groups. In addition, differences in the intercepts across the SES groups were examined to determine whether SES differences in EA persisted when taking into account PGS differences between children. Finally, in a within-family design, it was investigated whether DZ twins with the higher PGS (higher than their co-twins) scored higher on EA than their co-twins. This was done to examine if there was an association between a child’s PGS and EA independent from the effect of SES.

### Model comparison

Nested models were compared using log-likelihood difference tests. Under certain regularity conditions,^52^ twice the difference in log-likelihood between a model and a nested model with *k* fewer parameters is asymptotically distributed as chi-squared with *k* degrees of freedom.

### Reporting summary

Further information on research design is available in the Nature Research Reporting Summary linked to this article.

## Supplementary information

Reporting Summary

## Data Availability

Due to older versions of the informed consent not covering the public sharing of raw data, the data comprising this study are not deposited in a publicly available repository. However, the raw data files on parental SES and EA, as extracted from the Netherlands Twin Register (NTR) data repository at the start of the study (2016), the EA PGS data based on the current set of genotyped NTR individuals (2018) and the scripts used to run the analyses are available upon request from the corresponding author. The study comprised of (1) a sample that included all twin pairs with data available for zygosity, parental SES and EA (at least one of the twins) and (2) a sample that included all children with data available for EA PGS, parental SES and EA. The NTR is an ongoing longitudinal study which means that more data on parental SES, EA, and PGS are currently being collected and will become available over the years and could also be requested for the two samples in this study. These raw phenotype and genotype data may be accessed, upon approval of the data access committee, by contacting the NTR (ntr.fgb@vu.nl).

## Supplementary information

**Supplementary Information** accompanies the paper on the *npj Science of Learning* website (10.1038/s41539-019-0052-2).
